# Prediction of Drug-Target Interactions and Drug Repositioning via Network-Based Inference

**DOI:** 10.1371/journal.pcbi.1002503

**Published:** 2012-05-10

**Authors:** Feixiong Cheng, Chuang Liu, Jing Jiang, Weiqiang Lu, Weihua Li, Guixia Liu, Weixing Zhou, Jin Huang, Yun Tang

**Affiliations:** 1 Shanghai Key Laboratory of New Drug Design, School of Pharmacy, East China University of Science and Technology, Shanghai, China; 2 School of Business, East China University of Science and Technology, Shanghai, China; Stanford University, United States of America

## Abstract

Drug-target interaction (DTI) is the basis of drug discovery and design. It is time consuming and costly to determine DTI experimentally. Hence, it is necessary to develop computational methods for the prediction of potential DTI. Based on complex network theory, three supervised inference methods were developed here to predict DTI and used for drug repositioning, namely drug-based similarity inference (DBSI), target-based similarity inference (TBSI) and network-based inference (NBI). Among them, NBI performed best on four benchmark data sets. Then a drug-target network was created with NBI based on 12,483 FDA-approved and experimental drug-target binary links, and some new DTIs were further predicted. *In vitro* assays confirmed that five old drugs, namely montelukast, diclofenac, simvastatin, ketoconazole, and itraconazole, showed polypharmacological features on estrogen receptors or dipeptidyl peptidase-IV with half maximal inhibitory or effective concentration ranged from 0.2 to 10 µM. Moreover, simvastatin and ketoconazole showed potent antiproliferative activities on human MDA-MB-231 breast cancer cell line in MTT assays. The results indicated that these methods could be powerful tools in prediction of DTIs and drug repositioning.

## Author Summary

Study of drug-target interaction is an important topic toward elucidation of protein functions and understanding of molecular mechanisms inside cells. Traditional methods to predict new targets for known drugs were based on small molecules, protein targets or phenotype features. Here, we proposed a network-based inference (NBI) method which only used drug-target bipartite network topology similarity to infer new targets for known drugs. The performance of NBI outperformed the drug-based similarity inference and target-based similarity inference methods as well as other published methods. Via the NBI method five old drugs, namely montelukast, diclofenac, simvastatin, ketoconazole, and itraconazole, were identified to have polypharmacological effects on human estrogen receptors or dipeptidyl peptidase-IV with half maximal inhibitory or effective concentration from submicromolar to micromolar by *in vitro* assays. Moreover, simvastatin and ketoconazole showed potent antiproliferative activities on human MDA-MB-231 breast cancer cell line in MTT assays. The results indicated that the drug-target bipartite network-based inference method could be a useful tool for fishing novel drug-target interactions in molecular polypharmacological space.

## Introduction

Over the past decade, the rate of new chemical entities transferred to therapeutic agents has been significantly decreased [1]. Interestingly, this phenomenon is concurrent with the dominant assumption that the goal of drug discovery is to design exquisitely selective ligands against a single target. However, this ‘one gene, one drug, one disease’ paradigm was challenged in many cases, and the concept of polypharmacology was hence proposed for those drugs acting on multiple targets rather than one target [1]. For example, serotonin and serotonergic drugs not only bind to G protein-coupled receptors (GPCRs) such as 5-hydroxytryptamine receptors 1, 2 and 4–7 (5-HT_1,2,4–7_), but also might bind to an ion channel, i.e. 5-HT_3_ [2], [3]. Such polypharmacological features of drugs enable us to understand drug side effects or find their new uses, namely drug repositioning [4]. Some good examples are thalidomide, sildenafil, bupropion and fluoxetine [4],[5].

To date, several *in silico* methods have been developed to address the issues of drug-target interaction (DTI) prediction and drug repositioning [6]–[11]. The conventional methods can be either ligand-based or receptor-based. Ligand-based methods like quantitative structure-activity relationships (QSAR) and similarity search are very useful in this context. For example, Keiser *et al.* predicted new molecular targets for known drugs using chemical two-dimensional (2D) structural similarity, namely similarity ensemble approach [6], [7]. Twenty-three new DTIs were confirmed and five of which were potent with K_i_ values<100 nM. Recently, Humberto *et al.* developed a multi-target QSAR (mt-QSAR) classifier and built a web server for DTI prediction [8]. Receptor-based methods like reverse docking have also been applied in drug-target (DT) binding affinity prediction, DTI prediction and drug repositioning [9]–[11]. However, those methods could not be used for targets whose three-dimensional (3D) structures are unknown.

More recently, several network-based and phenotype-based methods were developed for such purposes. Yildirim *et al.* constructed a bipartite graph composed of US Food and Drug Administration (FDA)-approved drugs and proteins linked by DT binary associations [12]. This method quantitatively showed an overabundance of ‘follow-on’ drugs. Campillos *et al.* identified new DTIs using side-effect similarity [13]. They tested 20 of unexpected DTIs and validated 13 ones by *in vitro* binding assays. Iorio *et al.* predicted and validated new drug modes of action and drug repositioning from transcriptional responses [14]. Recently Butte group also reported two successful examples of drug repositioning based on public gene expression data [15], [16]. Furthermore, Yamanishi *et al.* developed a bipartite graph learning method to predict DTI by integrating chemical and genomic spaces [17]. Though high overall predictive accuracy was obtained in Yamanishi's work, the sensitivity was anomaly low and the method was not validated experimentally.

In this study, three inference methods were developed to predict new DTI: drug-based similarity inference (DBSI), target-based similarity inference (TBSI) and network-based inference (NBI), all derived from complex network theory [18]–[21]. Four benchmark data sets with known drugs targeting enzymes, ion channels, GPCRs, and nuclear receptors respectively, were used to assess the performance of the methods in comparison with literature reports. The best-performed method was then selected to create a drug-target network of FDA-approved and experimental drugs and to predict new DTIs subsequently. Some of the predictions were further validated by *in vitro* assays. This work would provide new powerful tools for DTI prediction and drug repositioning.

## Results

**Figure 1:**
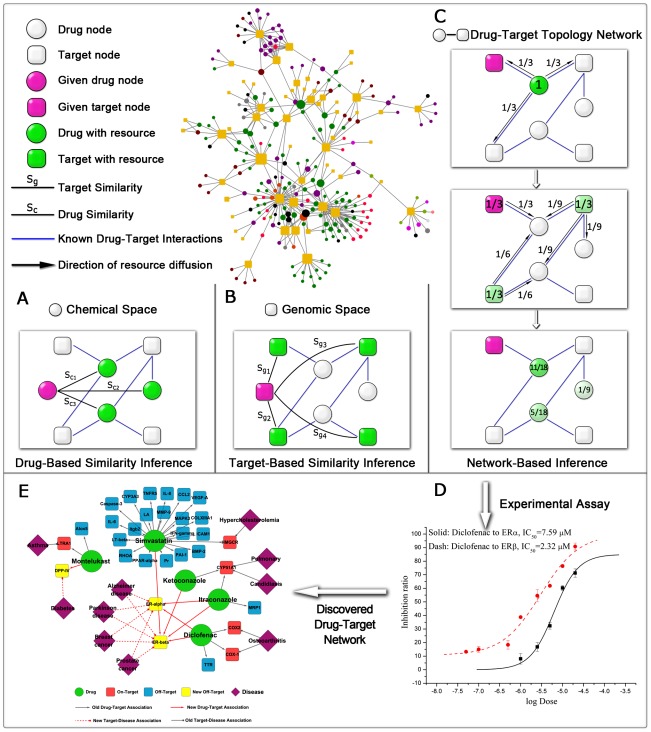
Schematic diagram of (A) drug-based similarity inference (DBSI), (B) target-based similarity inference (TBSI) and (C) network-based inference (NBI) methods. The entire workflow includes five steps: (i) collection of known drug-target interaction data and construction of bipartite drug-target graphs; (ii) calculation of drug-drug two dimensional structural similarity (S_C_), target-target genomic sequence similarity (S_g_) and drug-target topology network similarity; (iii) application of new methods in prediction of new drugs for a given target (pink square) or new targets for a given drug (pink circle); (iv) validation of new drug-target interactions by experimental assays (**D**); (v) visualization of experimental results using drug-target-disease associations network analysis (**E**). In **A–C**, given drug node (pink circle) denotes the drug which we want to predict new target for, given target node (pink square) denotes the target which we want to predict new drug for, drug with resource (green circle) denotes that this drug have resource, target with resource (green square) denotes that this target have resource, the more resource a node possesses, the darker the color is, blue edges denote the drug-target interactions with known experimental evidence, black arrows denote the resource diffusion direction. In **E**, green circle: drug node, red square: on-target node, blue square: off-target node, yellow square: new off-target node, violet square: disease node.

The methods developed here were derived from the recommendation algorithms of complex network theory, and proposed for DTI prediction and hence drug repositioning. In principle, the DBSI method (Figure 1A) is very similar to the item-based collaborative filtering method in recommendation algorithms [20], while TBSI (Figure 1B) is similar to the user-based collaborative filtering method [21]. Different from DBSI and TBSI, the NBI method (Figure 1C) only uses known DT bipartite network topology similarity to predict unknown DTI, which employs a process analogous to mass diffusion in physics across the DT network [18], [19]. In NBI method, predictive scores are calculated for each given drug (pink circle) and each unlinked target, and a recommendation list of drugs was created for a given target (pink square) in a descending order after the diffusion process.

### Performance of the methods on benchmark data sets

Four benchmark data sets were used to assess the performance of the methods. The data sets were named after four major drug targets, i.e. enzymes, ion channels, GPCRs, and nuclear receptors. At first, all known DTIs (Table S1) involved in the data sets were used to generate a DT bipartite network (Figure S1), in which a drug (circle) and a target (square) were connected if the target was known to the drug according to experimental evidence.

**Figure 2:**
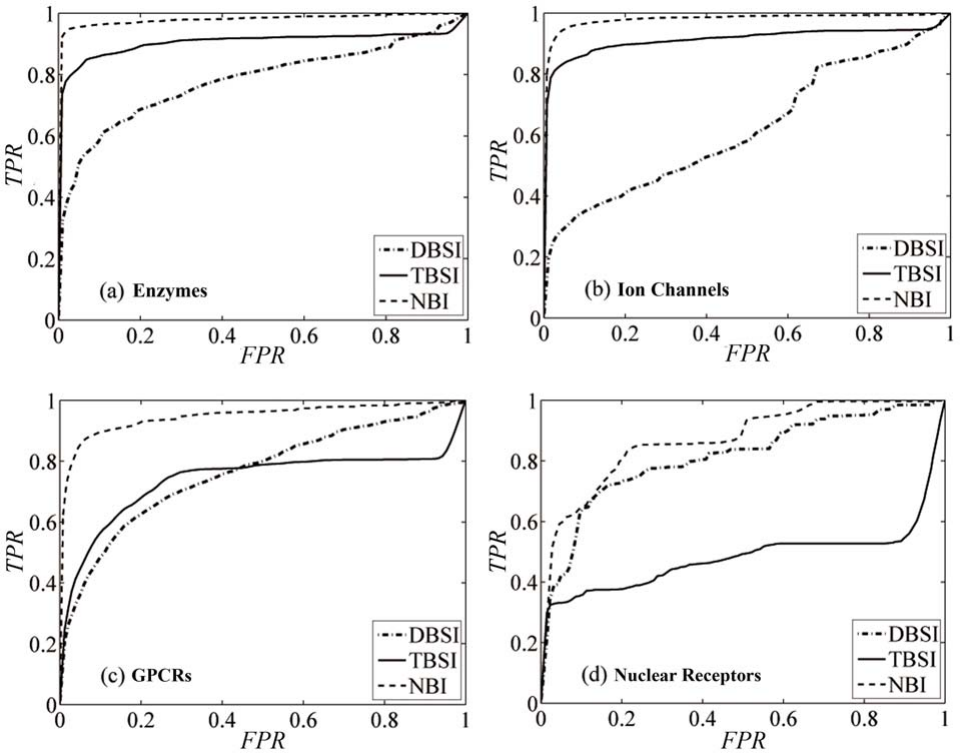
The receiver operating characteristic (ROC) curves of the three different methods to predict new known drugs for a given target on the four benchmark data sets by simulation 30 times of 10-fold cross validation test, (a) enzymes, (b) ion channels, (c) GPCRs and (d) nuclear receptors, drug-based similarity inference (DBSI): dot dash curve, target-based similarity inference (TBSI): solid curve, network-based inference (NBI): dash curve, FPR: false positive rate and TPR: true positive rate.

Figure 2 illustrated the receiver operating characteristic (ROC) curves calculated by the methods on the benchmark data sets using the 30 simulation times of 10-fold cross validation, from which it is easy to see that all methods performed well with high true positive rate (TPR) against low false positive rate (FPR) at any threshold. As shown in Figure 2, NBI always gave the best TPR values at any FPR value, suggesting that the NBI method would have the highest predictive ability among them. The average area under ROC curve (AUC) values of NBI method by the 30 simulation times of 10-fold cross validation were 0.975±0.006, 0.976±0.007, 0.946±0.019 and 0.838±0.087 for enzymes, ion channels, GPCRs and nuclear receptors, respectively (Table S2).

Figure S2 illustrated precision (*P*) as a function of predicted length (*L*) with different methods. For enzymes, ion channels and GPCRs, the curves from up to down were yielded for NBI (dash curve in the figure), TBSI (solid curve) and DBSI (dot dash curve) subsequently, which coincided with the performance of AUC. For nuclear receptors, the relation of the three curves was not so regular as in the former three data sets, which suggested that data completeness [22] should be important for DTI prediction because there were only 90 DTI pairs in the nuclear receptor data set and the average of known targets for a drug was less than 2 (Table S1). Figure S3 illustrated recall (*R*) as a function of *L* with different methods. The *R* value from NBI was much better than those from TBSI and DBSI (Table S3). It should be highlighted that the *R* value is the most important parameter in DTI modeling. A low *R* value indicated the low ability of a model to recognize known DTIs from complex DT networks.

### Prediction of drug-target interactions

**Figure 3:**
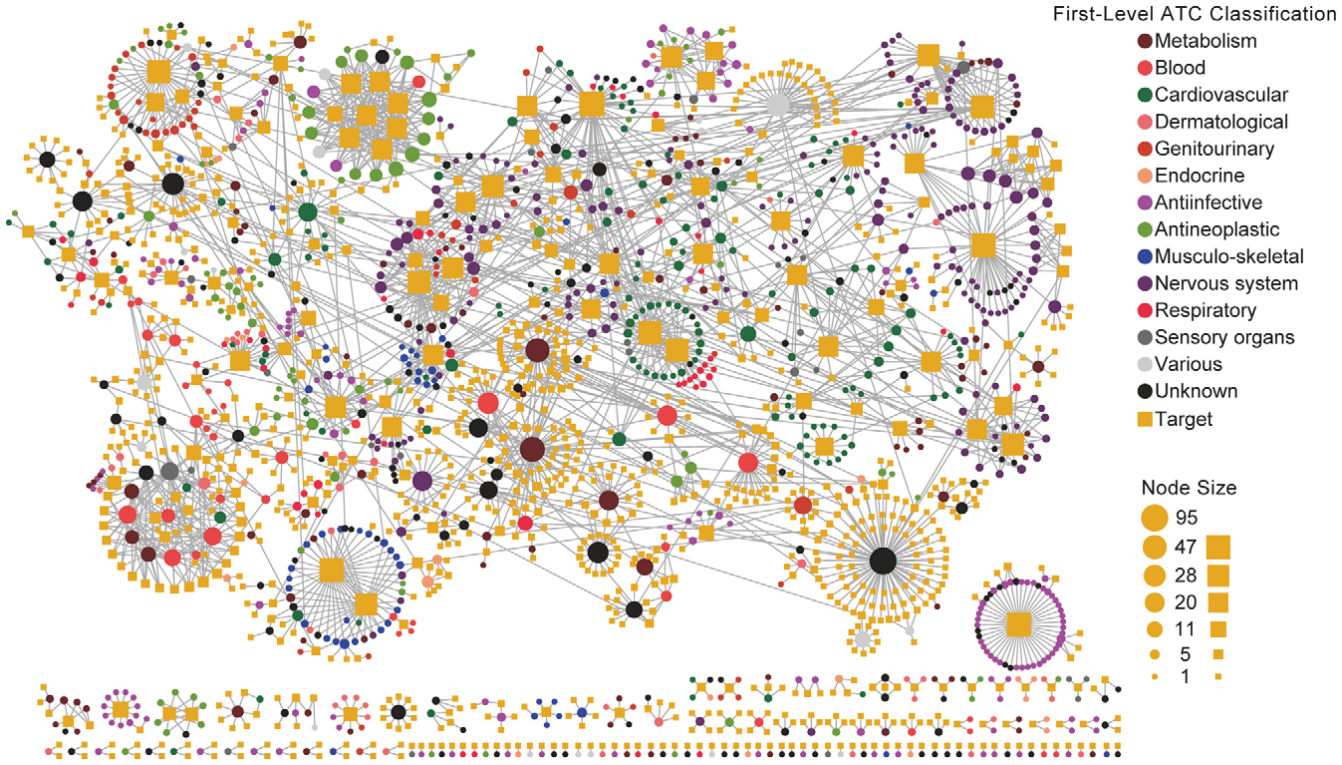
The drug–target (DT) bipartite network, in which a drug node (circle) and a target node (square) are connected to each other by grey edge if the target is annotated to have known experimental interactions with the drug in DrugBank. The DT network was generated using known FDA-approved small molecule DT interactions. The size of the drug node is the fraction of the number of targets that the drug linked in DrugBank. The size of the target node is the fraction of the number of drugs that the target linked in DrugBank. Color codes are given in the legend. Drug nodes (circles) are colored according to their Anatomical Therapeutic Chemical Classification. The graph was prepared by Cytoscape (http://www.cytoscape.org/).

At first, a DT bipartite network was constructed with known DTI data extracted from DrugBank [23]. As shown in Figure 3, there were obviously polypharmacological features for many approved drugs. For example, the promiscuous drug NADH was connected with 95 proteins, while the promiscuous target α_1A_ adrenergic receptor was linked with 52 drugs. This comprehensive mapping of pharmacological space enables us to predict new indications for old drugs by our methods.

NBI method was then used to predict new DTI in the DT bipartite network. To test the feasibility of NBI on DrugBank, the performance was assessed by the 30 simulation times of 10-fold cross validation. As shown in Figure S4, high AUC values of 0.865±0.009 and 0.849±0.012 were yielded with NBI for the approved drugs and the global data set containing approved and experimental drugs, respectively, which indicated that NBI method is valid for DrugBank.

In order to validate the predictions experimentally, one enzyme, DPP-IV, and two receptors, ERα and ERβ, were selected as the targets, just because the drug screening systems of these targets are available in our laboratory. By applying NBI method on the global DrugBank database, all new potential drugs targeted with DPP-IV, ERα and ERβ were predicted. Nine purchasable old drugs were selected from top 50 recommended potential DPP-IV inhibitors (Table S4), whereas 31 purchasable old drugs were selected from top 80 recommended potential ER ligands (Tables S5 and S6) for experimental assays.

### Experimental validation of drug repositioning

**Figure 4:**
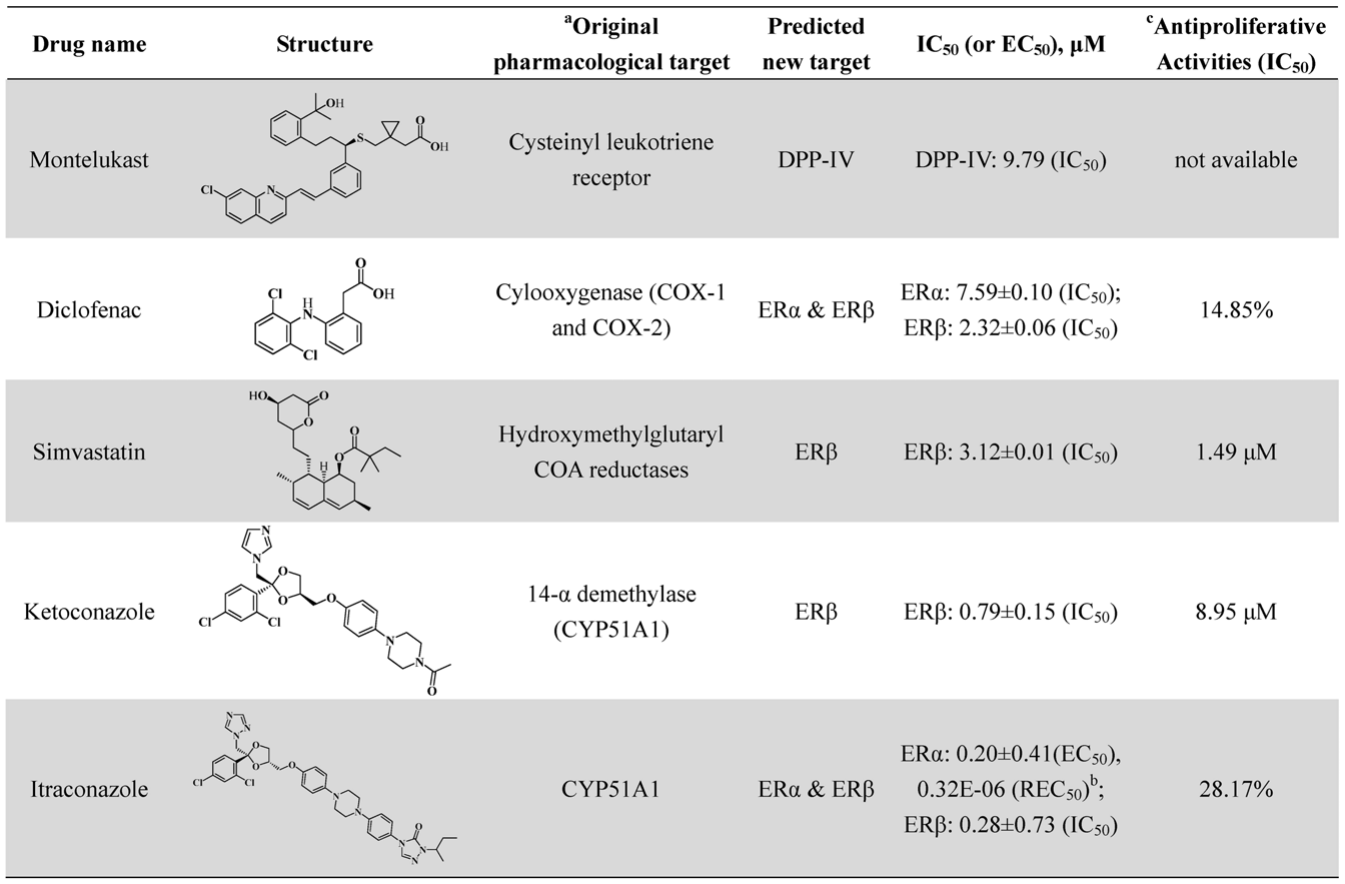
Predicted and bioassay results of new identified drug-target indications for five known approved drugs. Data shown are the mean for at least triplicate measurements. ^a^Original pharmacological target information was extracted from DrugBank (http://www.DrugBank.ca/). ^b^50% relatively effective concentration is the concentration of the tested chemical showing 50% of agonistic activity of the maximum activity of E2. REC_50_ provides the estrogenic activity relative to that of E2. ^c^Antiproliferative activities were assayed on human MDA-MB-231 breast cancer cell line by MTT assays. IC_50_: half maximal inhibitory concentration, EC_50_: half maximal effective concentration, ER: Estrogen Receptors, DPP-IV: dipeptidyl peptidase-IV.

**Figure 5:**
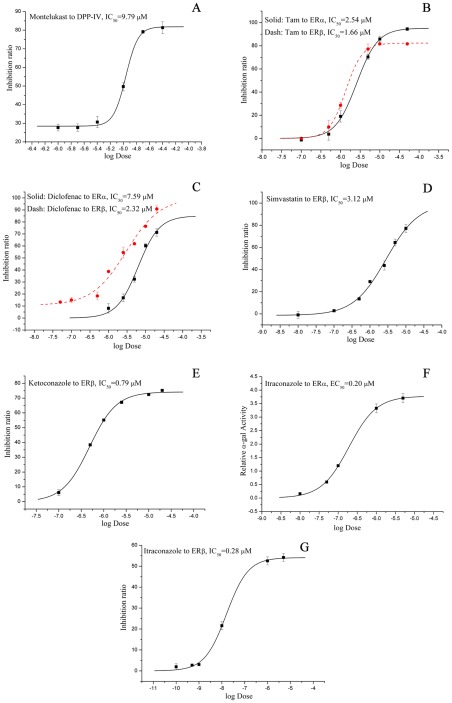
Dose-response curves of experimentally validated polypharmacology activities on estrogen receptor (ER) and dipeptidyl peptidase-IV (DPP-IV). Dose-response curves for inhibitive activation: montelukast to DPP-IV (A), for transcriptional activation: tamoxifen (Tam) to ERα (black solid line) and ERβ (red dash line) (B), diclofenac to ERα (black solid line) and ERβ (red dash line) (C), simvastatin to ERβ (D), ketoconazole to ERβ (E), itraconazole to ERα (F), itraconazole to ERβ (G). In A–G, error bars were presented as the mean 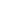
SD (standard deviation) of three duplicate determinations.

All the 40 old drugs were purchased and tested by *in vitro* assays accordingly. As shown in Figures 4 and 5, one approved drug, i.e. montelukast, was identified from the 9 purchased compounds as an unreported DPP-IV inhibitor with half maximal inhibitory concentration (IC_50_) = 9.79 µM. For ERα and ERβ, four approved drugs, namely diclofenac, simvastatin, ketoconazole, and itraconazole, were identified out of the 31 compounds as novel ER ligands with IC_50_ or half maximal effective concentration (EC_50_) values less than 10 µM. Itraconazole was a dual-profile compound, which showed agonistic activity with EC_50_ of 200 nM on ERα but a higher antagonistic activity with IC_50_ of 280 nM on ERβ than tamoxifen, a classical anti-breast cancer drug.

**Figure 6:**
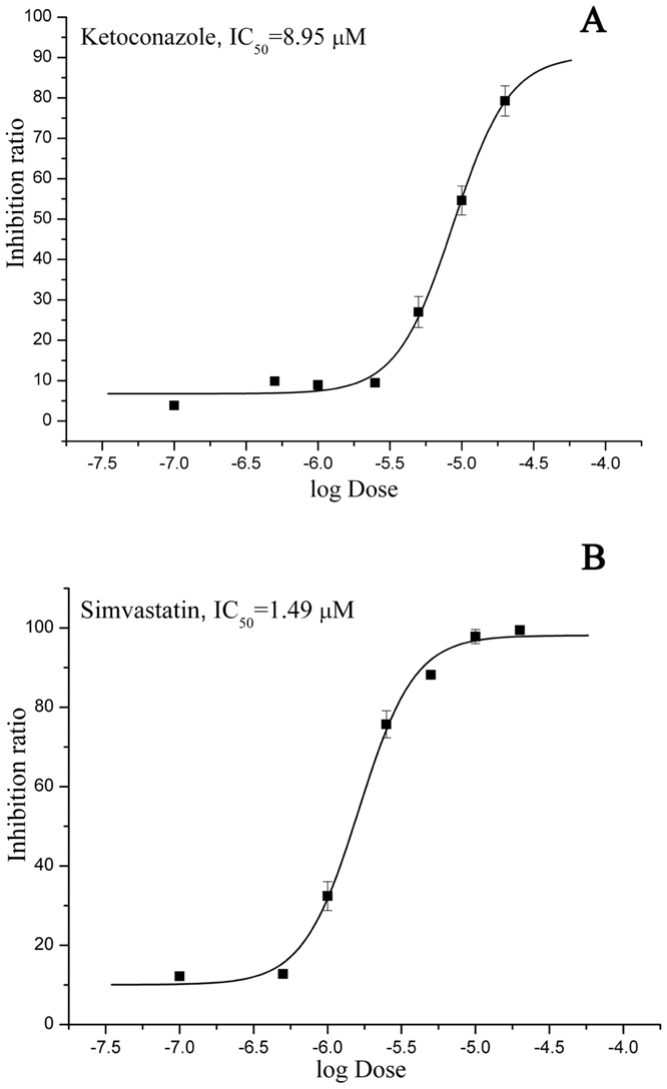
Dose-response curves of the antiproliferative potencies for ketoconazole (A) and simvastatin (B) on human MDA-MB-231 breast cancer cell lines by MTT assay. Error bars are presented as the 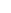
SD (standard deviation) of three duplicate determinations.

Moreover, the antiproliferative potencies of diclofenac, simvastatin, ketoconazole, and itraconazole were evaluated on human MDA-MB-231 breast cancer cell line by MTT assays. As shown in Figure 6, simvastatin and ketoconazole showed potent antiproliferative activities with IC_50_ values of 1.49 µM and 8.95 µM, respectively.

### Network visualization of validated drug-target interactions

**Figure 7:**
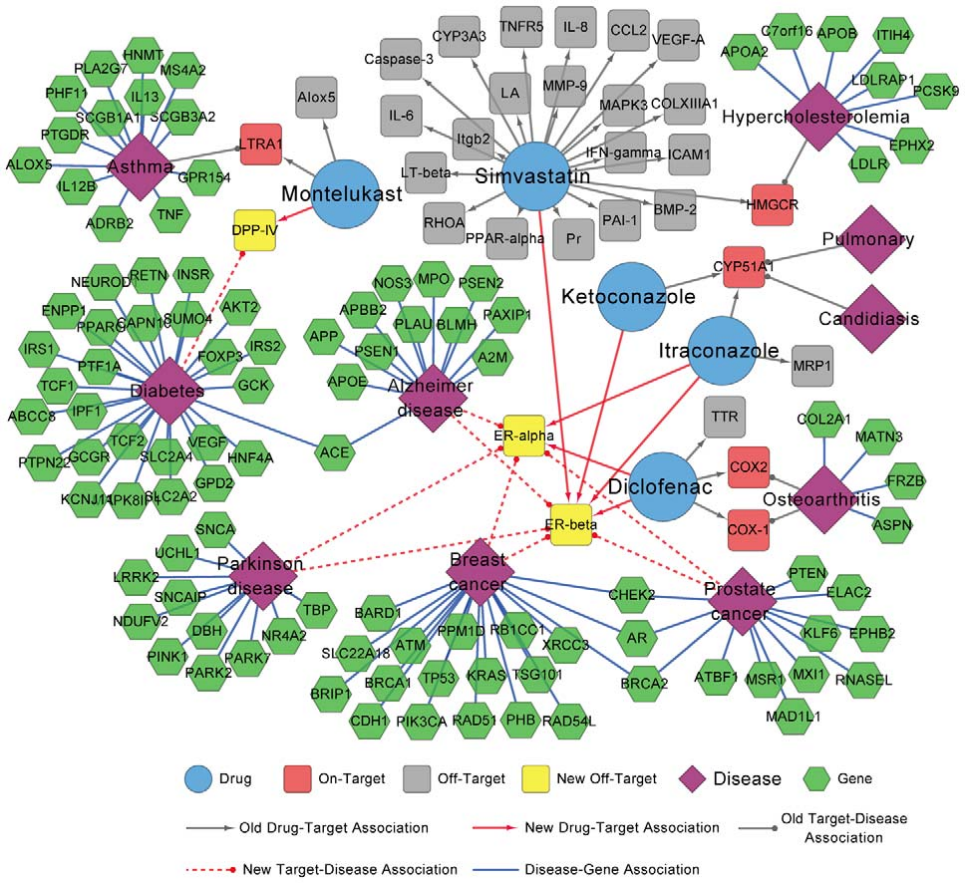
Discovered drug-target, target-disease and disease-gene associations network. Grey arrows denote the old drug-target interactions, grey edges denote the old target-disease associations and blue edges denote the known disease-gene associations, which were extracted from DrugBank, Online Mendelian Inheritance in Man (OMIM) Morbid Map and literature reports (The further data were given in Table S7). Red arrows among approved drug nodes (cyan circle) and target nodes (yellow squares) denote the new discovered drug-target interactions in this study. Red dotted edges denote new target-disease associations discovered in this study. Cyan circle: drug node, red square: on-target (Primary targets annotated in DrugBank), grey square: off-target, yellow square: new off-target (new discovered target for a given drug validated in this study), violet square: disease node, green regular hexagon: gene. The graph was prepared by Cytoscape (http://www.cytoscape.org/).

Network visualization of drug-target, target-disease and disease-gene associations could provide helpful information for discovery of new therapeutic indications or adverse effects of old drugs. As illustrated in Figure 7, where disease-related genes and disorder-disease gene associations (given in Table S7) were extracted from Online Mendelian Inheritance in Man (OMIM) Morbid Map [24], it is easy to see polypharmacological effects of the five old drugs (cyan). For example, simvastatin originally inhibits HMG-CoA reductase (on-target labeled with red square box) [23], [25], but it has more than 20 off-targets (gray square box) in Figure 7 [24]. In this study, simvastatin was validated to have antagonistic effects on ERβ with IC_50_ value at 3.12 µM and showed good antiproliferative activity on human MDA-MB-231 breast cancer cell line with IC_50_ value of 1.49 µM (Figures 5 and 6).

Although some drugs act by binding to specific proteins, most of FDA-approved drugs were developed without knowledge of molecular mechanisms responsible for their indicated diseases. For example, ketoconazole inhibits the production of testosterone, and has been used by urologists to treat refractory bone pain and impending neurologic injury in patients with advanced metastatic prostate cancer [26], [27], but the molecular mechanism is unknown. In this study, ketoconazole was found to selectively inhibit ERβ with IC_50_ value of 0.79 µM and showed good antiproliferative activity on human MDA-MB-231 breast cancer cell line with IC_50_ value of 8.95 µM, which indicated that ketoconazole may have more broad-spectrum anti-cancer indications with therapeutic effects of breast cancer in clinic.

## Discussion

### Comparison of the methods

In this study, three supervised inference methods, i.e. DBSI, TBSI and NBI, were developed to predict new DTI. Excellent performance was obtained for these methods on four benchmark data sets, which outperformed some methods reported elsewhere [17], [28], [29]. The essential difference of the three methods is the definition of similarity. DBSI is based on chemical 2D structural similarity, and TBSI is based on genomic sequence similarity, whereas NBI is only based on DT bipartite network topology similarity (Figure 1). The worse AUC values of DBSI on the benchmark data sets indicated that the prediction based on chemical structure similarity alone was poor (Figure 2). This may be caused by the redundancy in the similarity. For example, in the enzyme data set, though chemical structure similarity can present drug similarity very accurately, similar structures without binding to enzymes should be redundant to reduce the predictive accuracy. There is a similar redundancy problem in TBSI. Although NBI is the simplest one for ignoring structural information of drugs and targets, the prediction is the most reliable (see box plot in Figure S5). And NBI only used DTI topology network similarity for inferring new potential DTI, which did not need any 3D structural information of targets and drugs. Therefore, NBI performed better than DBSI, TBSI and other reverse docking methods [10], [11].

Recently, Hansen *et al.* created four features from gene-drug network and built a logistic classifier for drug-gene association prediction [30]. Although high predictive performance were obtained, an inherent problem in Hansen's work is that the negative drug-gene pairs were randomly constructed (selected on the basis of unknown drug-gene associations), which easily brought noise in a logistic classifier building by the inaccurate negative sample selection. Yamanishi *et al.* predicted new DTIs by integration of chemical and genomic spaces. Reasonable AUC value was obtained, but the *R* values were extremely poor, only 0.574, 0.271, 0.234 and 0.148 for enzymes, ion channels, GPCRs, and nuclear receptors respectively [17], and the predicted results were not validated experimentally. Compared with those reported methods, NBI only used the simple DT association information and yielded high predictive performance (*R* more than 0.9, Table S3). Chiang and Butte developed a guilt-by-association method for disease-gene association prediction and drug repositioning [31]. This method only used gene-disease linkage information. In present study, NBI takes fully advantage of the labeled and unlabeled information encoded in the full DT network topology (Figure 1), thereby simultaneously exploiting both topological and functional modularity.

### Potential application of NBI in drug repositioning

Usually there are two major methods for DTI prediction and drug repositioning: traditional drug discovery method, in which new drugs or hits are predicted for a certain target; and chemical biology method, where new potential targets are predicted for a given drug or chemical [17]. In this study, NBI method inherited the advantages of both methods. It can prioritize candidate drugs for a given target or prioritize candidate targets for a given drug simultaneously by personal recommendation [18], [19]. With matrix transposition, we could also prioritize new potential targets for a given drug. As shown in Figure S6, the high performance was yielded for our three methods in prediction of new candidate targets for a given drug, and NBI exhibited the highest predictive accuracy. Therefore, NBI could be a powerful tool in drug repositioning.

Since NBI only utilized known DTI information, for a new drug without known target information in the training set, NBI could not predict targets for this new drug. This is a weakness of the method. However, potential targets of a new drug can be predicted by integrating DBSI, TBSI and NBI together. We are actively developing new network inference method by integrating drugs, proteins and phenotype features based on diffusion theory [32]. Our methods could also be used in prediction of other biological networks, such as protein-protein interactions, drug-gene, gene-disease, and drug-disease networks, by integrating additional similarity measures among diseases, genes, and drugs [33]–[35].

### Polypharmacological features of new DPP-IV inhibitor

**Montelukast**, antagonist of cysteinyl leukotriene 1 receptor, was marketed in the US and other countries by Merck with the brand name Singulair®. Although Langlois *et al.* reported that montelukast regulates eosinophil protease activity through a leukotriene-independent mechanism recently [36], there is no report about its binding with DPP-IV so far. Herein, montelukast was predicted and validated as a new DPP-IV inhibitor with IC_50_ = 9.79 µM. Recently, Faul *et al.* found that oral administration of montelukast could change the weak level of Insulin in small scale clinical experiment [37]. Therefore, it is reasonable to deduce that montelukast might have new potential indication in anti-diabetic treatment via inhibiting DPP-IV (Figure 7). Comparing the structural similarity between montelukast and sitagliptin, a classical DPP-IV inhibitor, the Tanimoto similarity based on MACCS keys [38] was only 0.38, which confirmed that NBI could successfully predict novel structural skeleton molecules for a given target.

### Polypharmacological features of new ER ligands

**Diclofenac** is an acetic acid nonsteroidal antiinflammatory drug (NSAID) with analgesic and antipyretic properties, and widely used to treat pain, dysmenorrhea, ocular inflammation, and so on. In the past decades, the anti-inflammatory effects of diclofenac were thought to be linked with inhibition of both leukocyte migration and cyclooxygenase (COX-1 and COX-2), leading to the peripheral inhibition of prostaglandin synthesis [23]. Herein, we reported that diclofenac targeted ERα and ERβ with IC_50_ values of 7.59 and 2.32 µM, respectively for the first time (Figure 4). There were a few similar examples to show NSAIDs targeting nuclear receptors recently. Zhou *et al.* reported that sulindac could induce apoptosis by binding to retinoid X receptor α (RXRα) [39], while Lehmann *et al.* found that indomethacin could activate the peroxisome proliferator-activated receptors α and β [40]. There were also several reports to show that oral administration of ER ligands had neuroprotective and anti-inflammatory effects [41]. Since ERα and ERβ are widely expressed in several tissues including central nervous system, cardiovascular system, gastrointestinal system, and immune system [42], therefore the anti-inflammatory and neuroprotective effects of diclofenac might be resulted from the novel biological pathways of inhibition to ERα and ERβ (Figure 7).

**Simvastatin**, the methylated form of lovastatin, is an antilipemic agent which inhibits HMG-CoA reductase [23]. Here we identified that simvastatin could inhibit ERβ with IC_50_ = 3.12 µM. There is some evidence to support our finding. For example, Wolozin *et al.* reported that simvastatin was associated with a strong reduction in the incidence of dementia, Alzheimer's disease (AD) and Parkinson's disease (PD) [43], [44]; several studies proved that estrogen treatment was effective in many neurodegenerative disease models [41], [45]; and statins were also found to have inhibitory effects on the proliferation of human breast cancer cells [46]. Therefore, the strong reduction in the incidence of dementia and PD and the inhibitory effects of the proliferation of human breast cancer cells could be explained by the potential novel biological pathway of inhibition to ERβ by simvastatin in Figure 7.

**Ketoconazole** and **Itraconazole**, as 14-α demethylase (CYP51A1) inhibitors, are synthetic antifungal drugs [23] and could be used to treat refractory bone pain and neurologic injury in patients with advanced metastatic prostate cancer [26], [27]. In this study, both drugs were identified to bind to ERα and ERβ with IC_50_ or EC_50_ value less than 1 µM (Figure 5). 14-α demethylase and ER did not share any common features in structures or functions, but they were deduced to have the same ligands by NBI method. The data showed that the therapeutic effect of ketoconazole in prostate cancer could be explained by the selective inhibition of ERβ by ketoconazole.

In last decades, tissue- or subtype-selective ER modulators (SERM) showed great advantages in clinic due to less adverse side effects [47], [48]. As shown in Figure 4, ketoconazole selectively inhibit ERβ with IC_50_ = 0.79 µM, and it did not show any antagonistic or agonistic activity to ERα. However, itraconazole was a dual-profile compound, which showed agonistic activity on ERα but a higher antagonistic activity on ERβ than the classical anti-breast cancer drug tamoxifen (Figure 5). Both ketoconazole and itraconazole could serve as leads for the discovery of novel oral SERM.

## Materials and Methods

### Data preparation

#### Benchmark data sets

All DTI data in the benchmark data sets were collected from KEGG BRITE [49], BRENDA [50], SuperTarget [51] and DrugBank [23]. As listed in Table S1, the numbers of known drugs in each data set were 445, 210, 223 and 54; while the numbers of targets in the data sets were 664, 204, 95 and 26 for enzymes, ion channels, GPCRs and nuclear receptors, respectively. The corresponding numbers of known interactions were 2926, 1476, 635 and 90. Further description about the data sets can be found in the original paper [17].

#### DrugBank database

The DrugBank database (accessed on August 25, 2010) was downloaded from the website: http://www.drugbank.ca/ [23]. The initial database contained 6,796 drug entries including 1,437 FDA-approved drugs and 5,174 experimental drugs. Entries containing inorganic compounds, non-covalent complexes, biotechnology drugs and mixtures were excluded. The refined database contained 12,483 DTIs, among which 2,988 ones were based on FDA-approved drugs.

All data sets used in this study are available online: http://www.lmmd.org/database/dti/.

### Method description

Denoting the drug set as 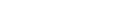 and target set as 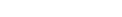, the DTI can be described as a bipartite DT graph 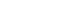, where 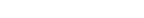. A link is drawn between 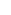 and 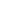 when the drug 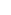 is associated with the target 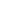. The DT bipartite network can be presented by an 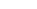 adjacent matrix 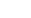, where 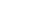 if 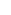 and 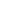 is linked, otherwise 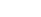.

#### Drug-based similarity inference (DBSI)

The basic idea of this method is: if a drug interacts with a target, then other drugs similar to the drug will be recommended to the target (Figure 1A). For a DT pair 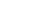, a linkage between 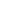 and 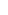 is determined by the following predicted score:
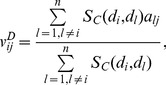
(1)where 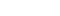 is 2D chemical similarity between drugs 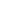 and 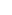, which was calculated by SIMCOMP [52] here.

#### Target-based similarity inference (TBSI)

The main idea of this method is: if a drug interacts with a target, then the drug will be recommended to other targets with similar sequences to the target (Figure 1B). For a DT pair 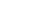, a linkage between 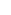 and 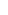 is determined by the following predicted score:
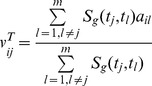
(2)Where 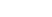 indicates the genomic sequence similarity between targets 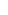 and 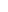, which was calculated by a normalized version of Smith-Waterman scores [17] here. All primary sequences of the targets were obtained from the KEGG GENES database.

#### Network-based inference (NBI)

Denoting 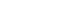, 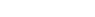 as the initial resource of drug 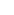, for a target 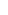, and 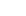 as the final resource of drug 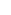. As shown in Figure 1C, for a general DT bipartite network, the final resource (score) 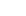 after two-step diffusion is:
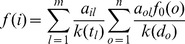
(3)where 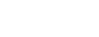 denotes the number of the targets that interact with 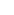, and 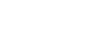 represents the number of the drugs that interact with 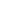.

The resource allocation process can be written as the matrix form as 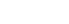, where, 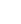 is the column vector of 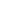, and 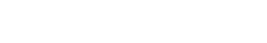 could be considered as the transfer matrix. 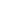 is the final configuration of resource on drugs.

For three methods, all 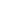
's unconnected drugs which are sorted in a descending order, constitute the recommendation list of the target 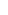. The drugs with the high predictive score in the list are more likely to interact with target 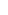.

### Performance assessment

To test the performance of the methods, 10-fold cross-validation approach was applied and each result was yielded by recalculating 30 times. For each data set, all the DTIs were randomly divided into 10 parts with equal size. Each part was taken in turn as the test set, while the remaining nine parts were served as the training set. With the randomly splitting, some targets (or drugs) may be just in the test set and the corresponding links without any information in the training set could not be predicted with the NBI method. Such links were not considered in the performance assessment.

Three parameters, AUC, precision (*P*) and recall (*R*), were calculated to assess the performance. The AUC value is obtained by calculating ranking score, which can be denoted as 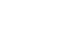, where 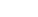 is the length of the recommendation list. And the average ranking score of the links in the test set is: 
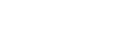
, where 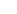 is the test set. And the AUC value is just equal to 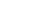. Since the links in the test set are actual DTIs, a good algorithm is expected to give good prediction for them, thus leading to large AUC.

*P* can be obtained from 
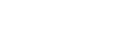
, where 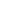 is the number of true positive predictions in the top 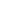 drugs in the recommendation list of target 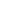. And *R* is defined as 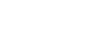, where 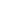 is the number of target 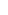
's missing links. Large *P* and *R* mean that more links in the gold standard interactions are predicted out.

### Prediction of drug-target interactions

Considering all DTI as known information, we calculated the recommendation list with top predictive scores via NBI method for all data sets. With the score ranking from high to low, the drugs in the topside of the list should be more likely to interact with the given targets, and the corresponding new DTIs were predicted. The full predicted lists of all data sets mentioned above are free available online: http://www.lmmd.org/database/dti/.

### Experimental validation

#### Compound purchase

Totally 40 purchasable approved drugs (Tables S4, S5, S6) were selected from the top recommendation lists for ERs and DPP-IV and purchased from the National Center for Drug Screening (http://www.screen.org.cn/), Shanghai, China.

#### Dipeptidyl peptidase-IV inhibition assay

The inhibitory effects of compounds on human recombinant DPP-IV was determined using a DPP-IV Drug Discovery Kit (Biomol, USA) according to the manufacturer's instructions. The activity of DPP-IV was detected in a Synergy™ 2 Multi-Mode Microplate Reader (BioTek) at an excitation wavelength of 380 nm and an emission wavelength of 460 nm. P32/98 (10 µM) was used as a positive compound. IC_50_ values were determined using the GraphPad Prism 4 software with three independent determinations.

#### Yeast two-hybrid system-based assay

To evaluate the agonistic or antagonistic activities of the compounds on ER, a yeast two-hybrid system was constructed by yeast co-transformation with pGBKT7-ERα/βLBD and pGADT7-SRC1 according to the lithium acetate method [53]. The combination plasmid pGBKT7-ERα/βLBD (amino acid residues 301–553 of ERα and 248–510 of ERβ) and pGADT7-SRC1 (amino acid residues 613–773) was prepared as described previously [54]. Butyl 4-(butyryloxy) benzoate functions as a new selective ERβ agonist and induces GLUT4 expression in CHO-K1 cells. After co-transforming the two constructs into yeast strain AH109, we successfully evaluated ER/SRC1 interactions by conducting a convenient α-galactosidase assay. Yeast transformants were incubated with either a control vehicle (DMSO) or the indicated compounds for 24 h in hERα/β agonist testing, and in antagonist assays 1 nM E2 was added. The α-galactosidase activity was then measured using *p*-nitrophenyl α-D-galactopyranoside as the substrate, according to the Clontech Yeast Protocol. The α-galactosidase activity was calculated according to equation 4:
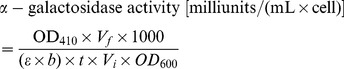
(4)where *t* is the elapsed time of incubation (min), *V_f_* is the final volume of assay (200 µL), *V_i_* is the volume of culture medium supernatant added (16 µL), OD_600_ is the optical density of overnight culture, and *ε×b* is the *p*-nitrophenol molar absorptivity at 410 nm×the light path (cm) = 10.5 mL/µmol.

#### MTT assays

Cell proliferation was quantified by MTT assay. MDA-MB-231 cells were seeded at a density of 1.5×10^4^ in a 96-well plate with DMEM/F12 supplemented with 10% charcoal stripped FBS without phenol red, and then incubated with the tested compounds in humidified air containing 5% CO_2_ at 37°C. After incubation for 24 h, 20 µL of 5 mg/mL MTT was added and incubated for another 4 h. Then the converted dye was dissolved in 100 µL of DMSO and the absorbance was measured at 570 nm.

## Supporting Information

Figure S1 The bipartite Drug–target network (DT network) graph for four benchmark data sets: enzymes (red), ion channels (orange), GPCRs (blue), nuclear receptors (black). Circles and rectangles correspond to drug and target nodes, respectively. A link is placed between a drug node and a target node if the protein is a known target of that drug. The size of the drug node is the fraction of the number of targets that the drug have with known experimental evidence. The size of the target node is the fraction of the number of drugs that the target have with known experimental evidence. The graph was prepared by Cytoscape (http://www.cytoscape.org/).
(TIF)

Figure S2The precision (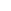) *versus* the predicted drugs length (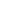) with the three different methods by 30 simulation times of 10-fold cross-validation test to predict new approved drugs to a given target (protein) for four benchmark data sets: (a) enzymes, (b) ion channels, (c) GPCRs and (d) nuclear receptors, (e) the log-log plot of *P* versus 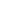 for the enzyme data. DBSI: Drug-Based Similarity Inference (dot dash curve), TBSI: Target-Based Similarity Inference (solid curve), NBI: Network-based Inference (dash curve).(TIF)

Figure S3The recall (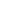) *versus* the predicted drugs length (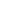) with the three different methods by 30 simulation times of 10-fold cross-validation test to predict new approved drugs to a given target (protein) for four benchmark data sets: (a) enzymes, (b) ion channels, (c) GPCRs, (d) nuclear receptors. DBSI: Drug-Based Similarity Inference (dot dash curve), TBSI: Target-Based Similarity Inference (solid curve), NBI: Network-based Inference (dash curve).(TIF)

Figure S4The performance of the network-based inference (NBI) method on the DrugBank data sets by 30 simulation times of 10-fold cross-validation test. (a) the receiver operating characteristic (ROC) curve, (b) precision (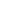) *versus* the predicted drugs length (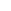), (c) recall (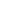) *versus* the predicted drugs length (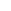), approved: data set of approved small molecular drugs in DrugBank, global: data set of approved and experimentally investigated small molecular drugs in DrugBank, FPR: false positive rate and TPR: true positive rate.(TIF)

Figure S5The box-plot of recalls (with the prediction list length 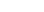) in the case of predicting new approved drugs for a given target by 30 simulation times of 10-fold cross-validation test. The green dash are plotted to distinguish the data sets, and three different methods are marked on the figure. DBSI: Drug-Based Similarity Inference, TBSI: Target-Based Similarity Inference, NBI: Network-based Inference, R: recall.(TIF)

Figure S6 The receiver operating characteristic (ROC) curve with the three different methods by 30 simulation times of 10-fold cross-validation test to predict new targets to a given drug, testing on four benchmark data sets: (a) enzymes, (b) ion channels, (c) GPCRs and (d) nuclear receptors. DBSI: Drug-Based Similarity Inference (dot dash curve), TBSI: Target-Based Similarity Inference (solid curve), NBI: Network-based Inference (dash curve).
(TIF)

Table S1 Statistic results of all known drug-target interaction (DTI) data sets used in this study.
(PDF)

Table S2 The performance of the area under receiver operating characteristic (AUC) for four benchmark data sets using three different methods by simulation 30 times of 10-fold cross validation test.
(PDF)

Table S3 Recall on the valid recommendation list length for all data sets using the NBI method by simulation 30 times of 10-fold cross validation test.
(PDF)

Table S4 The inhibitory activities of 9 approved drugs on dipeptidyl peptidase-IV.
(PDF)

Table S5 The agonistic and antagonistic activities of approved drugs for estrogen receptor α.
(PDF)

Table S6 The agonistic and antagonistic activities of approved drugs for estrogen receptor β.
(PDF)

Table S7 The detailed description of drug-gene-disease associations for five approved drugs (which were extracted from DrugBank and Online Mendelian Inheritance in Man (OMIM) Morbid Map on May, 2011).
(PDF)
