# Supplementary figures and images for: Prediction of Drug-Target Interactions and Drug Repositioning via Network-Based Inference
Source: PLoS Comput Biol. 2012 May 10;8(5):e1002503. doi: 10.1371/journal.pcbi.1002503 (PMC3349722; doi:10.1371/journal.pcbi.1002503)

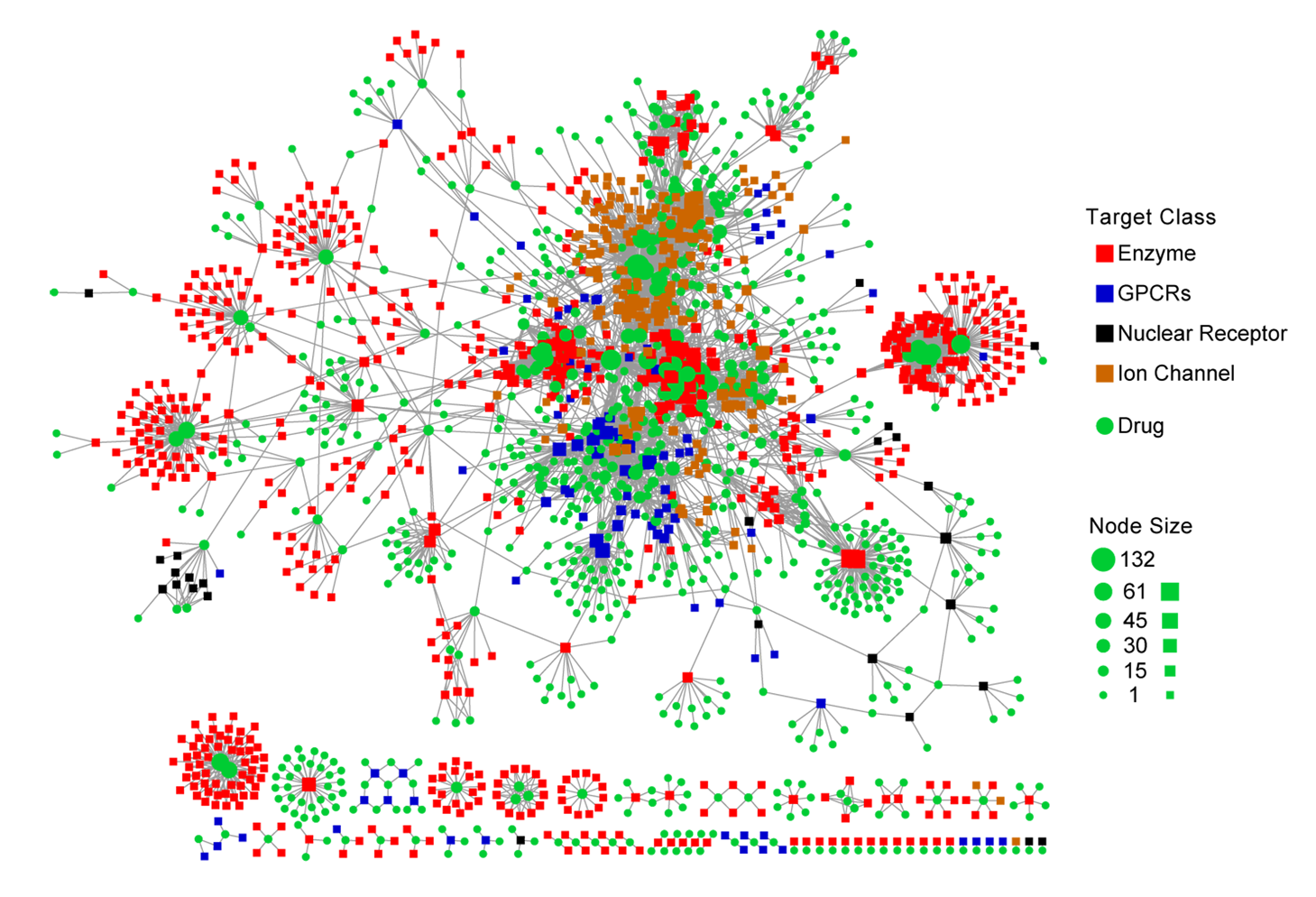

Supplement: Figure S1 — The bipartite Drug–target network (DT network) graph for four benchmark data sets: enzymes (red), ion channels (orange), GPCRs (blue), nuclear receptors (black). Circles and rectangles correspond to drug and target nodes, respectively. A link is placed between a drug node and a target node if the protein is a known target of that drug. The size of the drug node is the fraction of the number of targets that the drug have with known experimental evidence. The size of the target node is the fraction of the number of drugs that the target have with known experimental evidence. The graph was prepared by Cytoscape (http://www.cytoscape.org/). (TIF) [file pcbi.1002503.s001.tif]

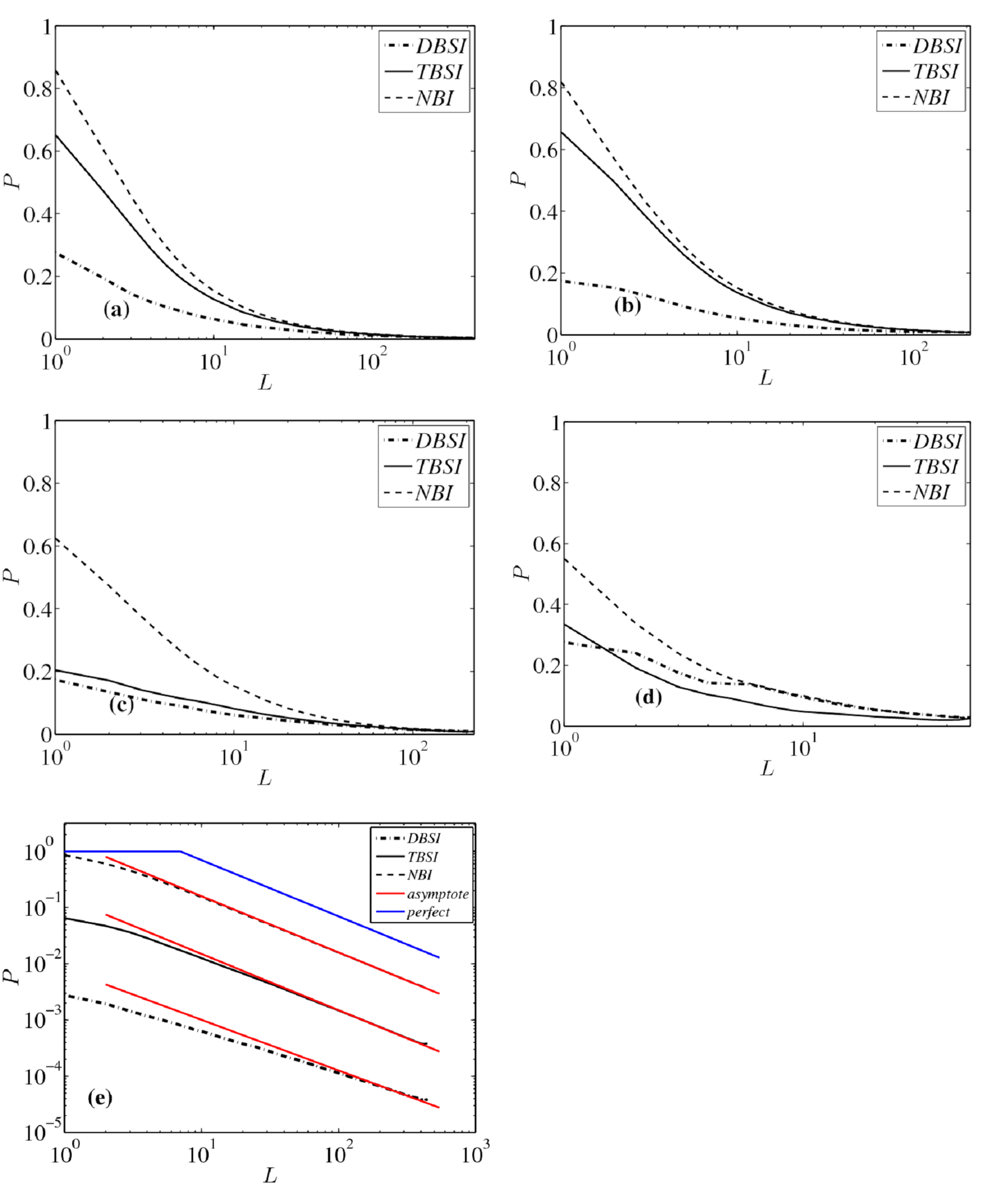

Supplement: Figure S2 — The precision () versus the predicted drugs length () with the three different methods by 30 simulation times of 10-fold cross-validation test to predict new approved drugs to a given target (protein) for four benchmark data sets: (a) enzymes, (b) ion channels, (c) GPCRs and (d) nuclear receptors, (e) the log-log plot of P versus for the enzyme data. DBSI: Drug-Based Similarity Inference (dot dash curve), TBSI: Target-Based Similarity Inference (solid curve), NBI: Network-based Inference (dash curve). (TIF) [file pcbi.1002503.s002.tif]

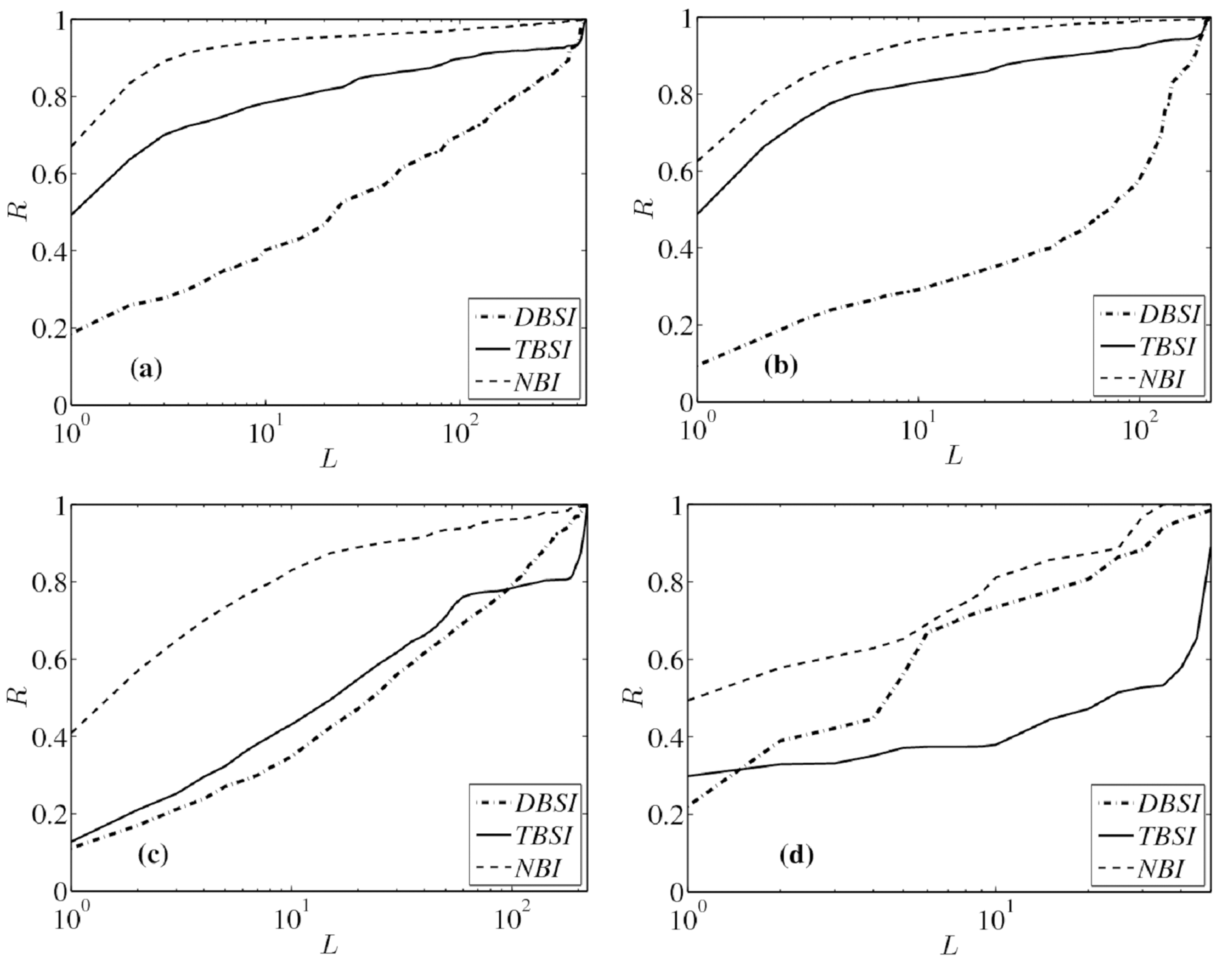

Supplement: Figure S3 — The recall () versus the predicted drugs length () with the three different methods by 30 simulation times of 10-fold cross-validation test to predict new approved drugs to a given target (protein) for four benchmark data sets: (a) enzymes, (b) ion channels, (c) GPCRs, (d) nuclear receptors. DBSI: Drug-Based Similarity Inference (dot dash curve), TBSI: Target-Based Similarity Inference (solid curve), NBI: Network-based Inference (dash curve). (TIF) [file pcbi.1002503.s003.tif]

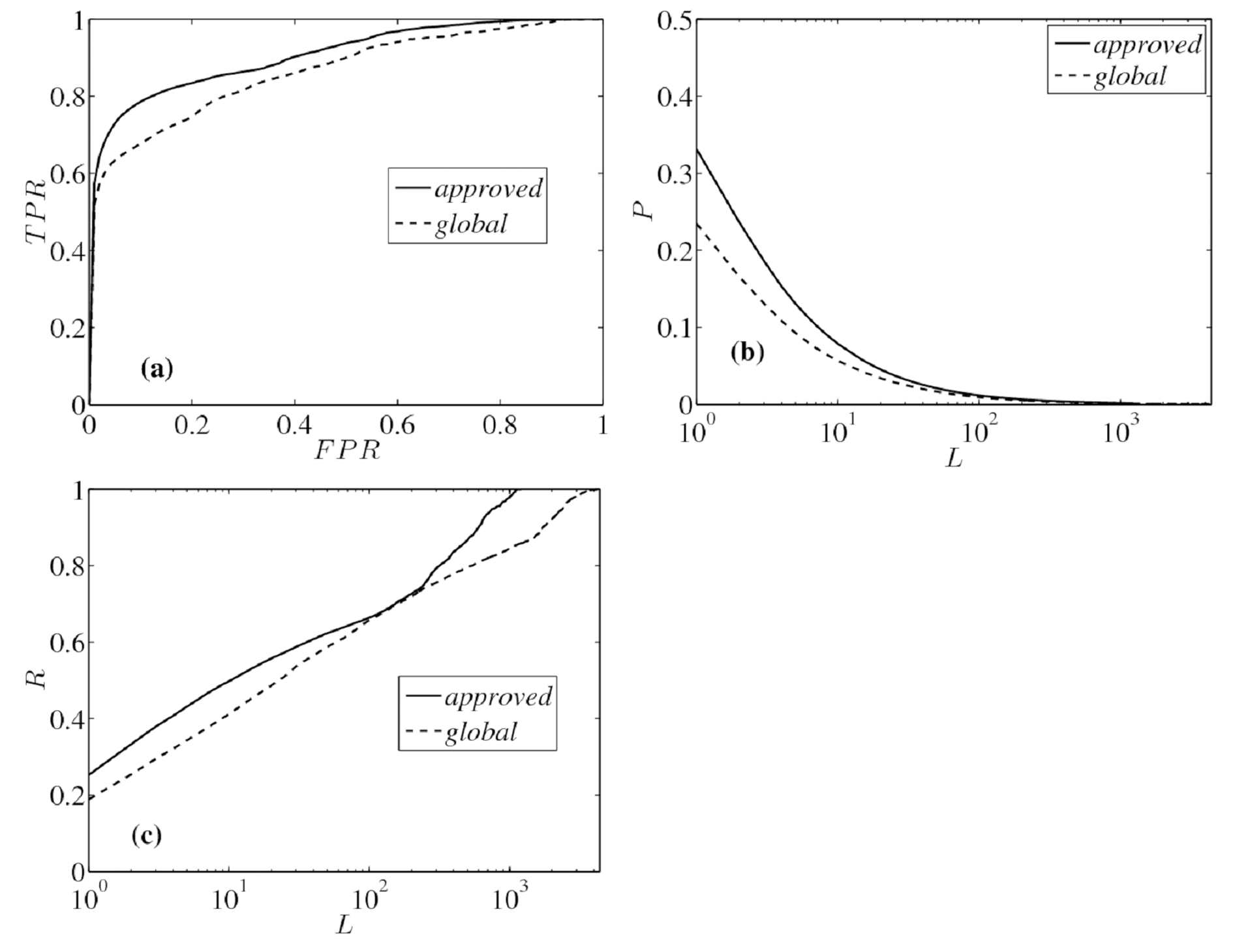

Supplement: Figure S4 — The performance of the network-based inference (NBI) method on the DrugBank data sets by 30 simulation times of 10-fold cross-validation test. (a) the receiver operating characteristic (ROC) curve, (b) precision () versus the predicted drugs length (), (c) recall () versus the predicted drugs length (), approved: data set of approved small molecular drugs in DrugBank, global: data set of approved and experimentally investigated small molecular drugs in DrugBank, FPR: false positive rate and TPR: true positive rate. (TIF) [file pcbi.1002503.s004.tif]

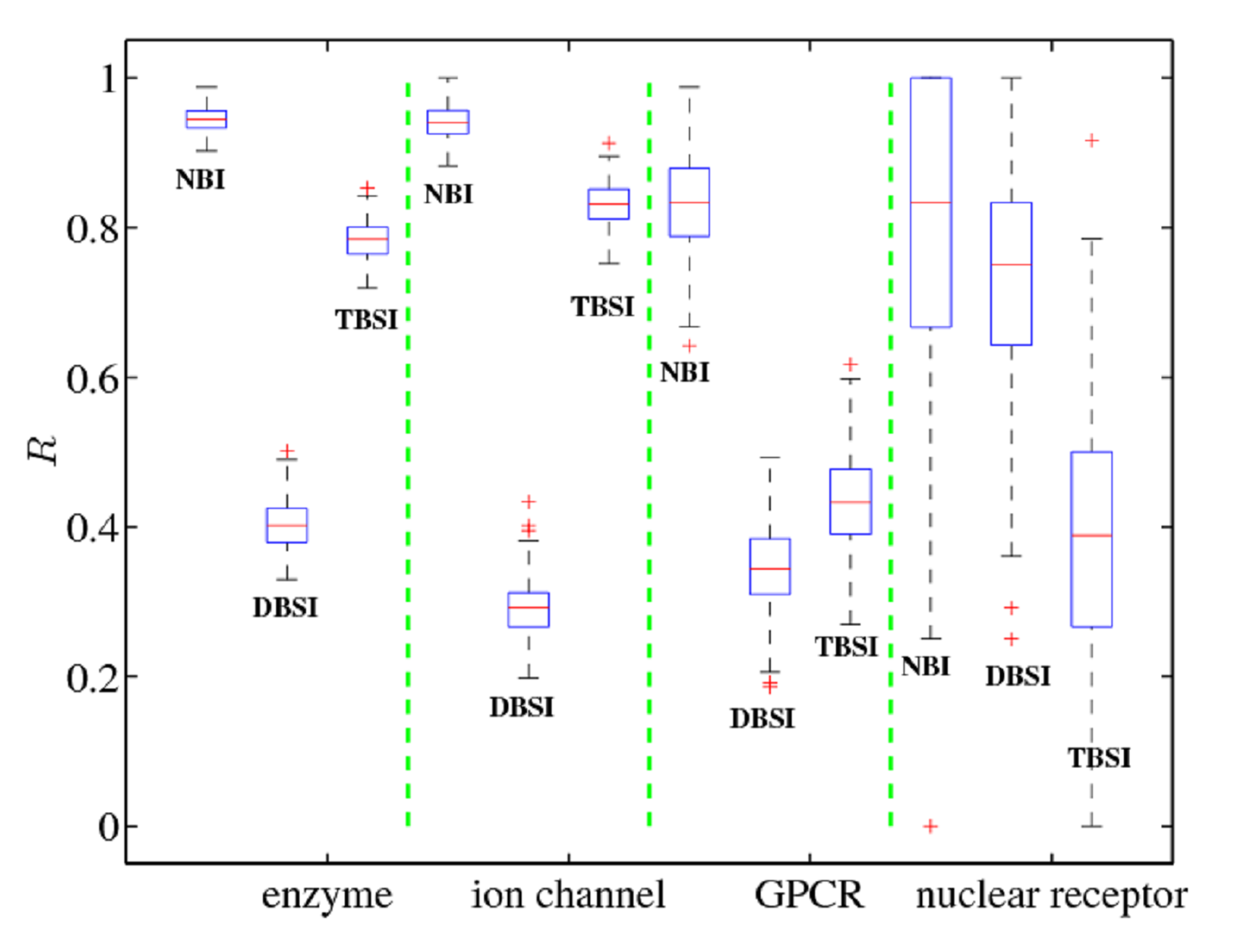

Supplement: Figure S5 — The box-plot of recalls (with the prediction list length ) in the case of predicting new approved drugs for a given target by 30 simulation times of 10-fold cross-validation test. The green dash are plotted to distinguish the data sets, and three different methods are marked on the figure. DBSI: Drug-Based Similarity Inference, TBSI: Target-Based Similarity Inference, NBI: Network-based Inference, R: recall. (TIF) [file pcbi.1002503.s005.tif]

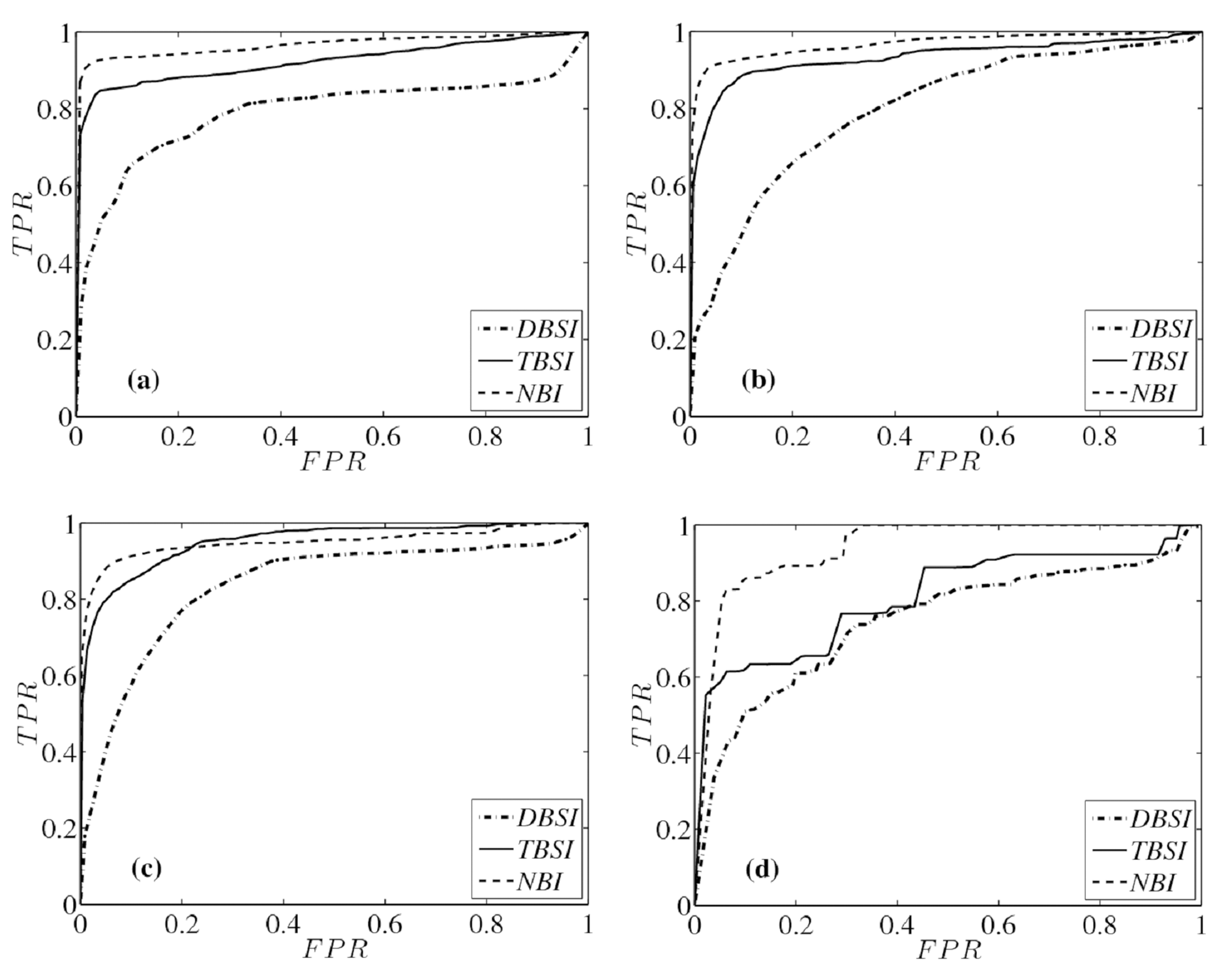

Supplement: Figure S6 — The receiver operating characteristic (ROC) curve with the three different methods by 30 simulation times of 10-fold cross-validation test to predict new targets to a given drug, testing on four benchmark data sets: (a) enzymes, (b) ion channels, (c) GPCRs and (d) nuclear receptors. DBSI: Drug-Based Similarity Inference (dot dash curve), TBSI: Target-Based Similarity Inference (solid curve), NBI: Network-based Inference (dash curve). (TIF) [file pcbi.1002503.s006.tif]
