# Supplementary material for: Prediction of Drug-Target Interactions and Drug Repositioning via Network-Based Inference
Source: PLoS Comput Biol. 2012 May 10;8(5):e1002503. doi: 10.1371/journal.pcbi.1002503 (PMC3349722; doi:10.1371/journal.pcbi.1002503)
Supplement: Table S1 — Statistic results of all known drug-target interaction (DTI) data sets used in this study. (PDF) [file pcbi.1002503.s007.pdf]

SUPPLEMENTARY MATERIAL

Prediction of Drug-Target Interactions and Drug Repositioning via Network-Based Inference

Feixiong Cheng[†,§], Chuang Liu[‡,§], Jing Jiang[†], Weiqiang Lu[†], Weihua Li[†], Guixia Liu[†], Weixing Zhou[‡,*], Jin Huang[†,*], and Yun Tang[†,*]

[§]These authors contributed equally to this work.

*To whom correspondence should be addressed.

For W. Zhou. Tel.: +86-21-6425-3634; Fax: +86-21-6425-3152. E-mail: wxzhou@ecust.edu.cn

For J. Huang. Tel.: +86-21-6425-3681; Fax: +86-21-6425-3681; E-mail: huangjin@ecust.edu.cn

For Y. Tang. Tel: +86-21-6425-1052; Fax, +86-21-6425-3651. E-mail: ytang234@ecust.edu.cn

Table S1 Statistic of all known drug-target interactions network used in this study.

data set	enzyme	ion channel	GPCRs	nuclear receptor	Approved	Global
N_d	445	210	223	54	1,120	4,398
N_t	664	204	95	26	1,112	3,801
N_i	2,926	1,476	635	90	2,988	12,483
Sparsity (%)	1.02	3.45	3.0	6.41	0.24	0.075

N_d is the number of the drugs, N_t is the number of the targets, N_i is the total number of the drug –target interactions, and sparsity is the proportion of the known drug-target interactions over all the possible interactions, approved: data set of approved small molecular drugs in DrugBank, global: data set of approved and experimentally investigated small molecular drugs in DrugBank.
