# Supplementary material for: Prediction of Drug-Target Interactions and Drug Repositioning via Network-Based Inference
Source: PLoS Comput Biol. 2012 May 10;8(5):e1002503. doi: 10.1371/journal.pcbi.1002503 (PMC3349722; doi:10.1371/journal.pcbi.1002503)
Supplement: Table S2 — The performance of the area under receiver operating characteristic (AUC) for four benchmark data sets using three different methods by simulation 30 times of 10-fold cross validation test. (PDF) [file pcbi.1002503.s008.pdf]

Table S2. The mean AUC for four benchmark datasets by 30 simulation times of 10-fold cross-validation test.

Methods	enzyme	Ion channel	GPCRs	Nuclear receptor
DBSI	0.781±0.018	0.709±0.025	0.755±0.033	0.789±0.084
TBSI	0.903±0.016	0.905±0.018	0.745±0.044	0.528±0.139
NBI	0.975±0.006	0.976±0.007	0.946±0.019	0.838±0.087

The AUC (ROC score) is the area under the receiver operating characteristic (ROC) curve. DBSI: Drug-Based Similarity Inference, TBSI: Target-Based Similarity Inference, NBI: Network-based Inference. The mean ± SD (standard deviation) was yielded by 30 simulation times of 10-fold cross-validation test.
