# Supplementary material for: Prediction of Drug-Target Interactions and Drug Repositioning via Network-Based Inference
Source: PLoS Comput Biol. 2012 May 10;8(5):e1002503. doi: 10.1371/journal.pcbi.1002503 (PMC3349722; doi:10.1371/journal.pcbi.1002503)
Supplement: Table S3 — Recall on the valid recommendation list length for all data sets using the NBI method by simulation 30 times of 10-fold cross validation test. (PDF) [file pcbi.1002503.s009.pdf]

Table S3. Recall on the valid recommendation list length for all data sets with the network-based inference (NBI) method by 30 simulation times of 10-fold cross-validation test.

Datasets	Enzyme	Ion Channel	GPCRs	Nuclear Receptor	Approved	Global
Recall	0.935	0.981	0.948	0.851	0.741	0.736

Approved: data set of approved small molecular drugs in DrugBank, global: data set of approved and experimentally investigated small molecular drugs in DrugBank.
