# Supplementary material for: Prediction of Drug-Target Interactions and Drug Repositioning via Network-Based Inference
Source: PLoS Comput Biol. 2012 May 10;8(5):e1002503. doi: 10.1371/journal.pcbi.1002503 (PMC3349722; doi:10.1371/journal.pcbi.1002503)
Supplement: Table S4 — The inhibitory activities of 9 approved drugs on dipeptidyl peptidase-IV. (PDF) [file pcbi.1002503.s010.pdf]

Table S4. The inhibitive activities profiles of nine approved drugs for dipeptidyl peptidase-IV (DPP-IV) enzyme inhibition assay. The inhibitory effects of compounds on human recombinant DPP4 was determined using a DPP-IV Drug Discovery Kit (Biomol, USA) according to the manufacturer's instructions. The DPP-IV Drug Discovery Kit is a complete assay system designed to screen DPP4 small molecule inhibitors. The fluorimetric assay is based on the cleavage of 7-amino-4-methylcoumarin (AMC) moiety from peptide substrate (H-Gly-Pro-AMC), which increases its fluorescence intensity at 460 nm. Compounds dilutions were prepared from stock in DMSO and diluted with assay buffer for inhibition assay. A 40 μL solution of DPP-IV enzyme in assay buffer was incubated with 10 μL of various concentrations of test compound solutions for 10 min at room temperature. The reaction was initiated by the addition of 50 μL of H-Gly-Pro- AMC with a final concentration of 5 μM. The activity of DPP4 was detected in a Synergy™ 2 Multi-Mode Microplate Reader (BioTek) at an excitation wavelength of 380 nm and an emission wavelength of 460 nm. P32/98 (10 μM) was used as a positive compound. IC_{50} values were determined by using the GraphPad Prism 4 software with three independent determinations.

DrugBank ID & Drug Name	Inhibition Ratio (%) to DPP-IV	IC_{50} (μM)
DB00622 (Nicardipine)	21.24	---
DB00227 (Lovastatin)	9.49	---
**DB00471 (Montelukast)**	**49.7**	**9.79**
DB01026 (Ketoconazole)	14.95	---
DB00641 **(**Simvastatin**)**	16.27	---
DB01259 (Lapatinib)	39.64	---
DB01132 (Pioglitazone)	18.63	---
DB00564 (Carbamazepine)	19.67	---
DB01241 (Gemfibrozil)	17.35	---
Positive Compounds (P32/98)	98.38（2μM）	98.38（2μM）
