# Supplementary material for: Prediction of Drug-Target Interactions and Drug Repositioning via Network-Based Inference
Source: PLoS Comput Biol. 2012 May 10;8(5):e1002503. doi: 10.1371/journal.pcbi.1002503 (PMC3349722; doi:10.1371/journal.pcbi.1002503)
Supplement: Table S5 — The agonistic and antagonistic activities of approved drugs for estrogen receptor α. (PDF) [file pcbi.1002503.s011.pdf]

Table S5. The agonistic and antagonistic activities profiles of approved small drugs for estrogen receptor α by yeast two-hybrid system-based assay. To evaluate the agonistic or antagonistic activities of the compounds, a yeast two-hybrid system for ER was constructed by yeast co-transformation with pGBKT7-ERα/βLBD and pGADT7-SRC1 according to the lithium acetate method. The detailed description can be found in the section of **Material and methods**. The concentration of compounds is 10 μM with DMSO solution.

DrugBank ID & Drug Name	Agonistic activities (10 μM）	Inhibitive ratio % (10 μM)
DMSO	1	0
E2 (1 nM)	13.07	--
DB00622 (Nicardipine)	0.962	15.21
DB01100 (Pimozide)	0.955	4.58
DB00836 (Loperamide)	0.934	6.18
DB01124 (Tolbutamide)	0.966	23.77
DB01085 (Pilocarpine HCl)	1.008	7.72
DB00501 (Cimetidine)	1.018	2.4
DB00909 (Zonisamide)	0.97	0.67
DB01200 (Bromocriptine mesylate)	1.034	3.36
DB01026 (Ketoconazole)	1.05	42.91
DB00752 (Tranylcypromine)	0.988	4.3
**DB00641 (Simvastatin)**	1.38	**60.4**
DB01259 (Lapatinib)	0.995	14.01
DB01217 (Anastrozole)	0.965	0
DB01128 (Bicalutamide)	1.007	11.66

Table S5. Continue…

DB00357 (Dl-aminoglutethimide)	0.956	0
**DB00586 (Diclofenac, Na)**	1.046	**63.49**
DB00741 (Hydrocortisone)	0.959	0.7
**DB01167 (Itraconazole)**	**4.132**	**8.19**
DB00834 (Mifepristone)	1.463	0
