# Supplementary material for: Prediction of Drug-Target Interactions and Drug Repositioning via Network-Based Inference
Source: PLoS Comput Biol. 2012 May 10;8(5):e1002503. doi: 10.1371/journal.pcbi.1002503 (PMC3349722; doi:10.1371/journal.pcbi.1002503)
Supplement: Table S6 — The agonistic and antagonistic activities of approved drugs for estrogen receptor β. (PDF) [file pcbi.1002503.s012.pdf]

Table S6. The agonistic and antagonistic activities profiles of approved small drugs for estrogen receptor β (ERβ) by yeast two-hybrid system-based assay. To evaluate the agonistic or antagonistic activities of the compounds, a yeast two-hybrid system for ER was constructed by yeast co-transformation with pGBKT7-ERα/βLBD and pGADT7-SRC1 according to the lithium acetate method. The detailed description can be found in the section of **Material and methods**. The concentration of compounds is 10 μM with DMSO solution.

Drugbank ID & Compound name	Agonistic activities（10 μM）	Inhibitive Ratio%（10 μM）
DMSO	1	0
E2 (1 nM)	5.77	---
Melatonin (DB01065)	0.914	0
DB01006 (Letrozole)	0.846	0
DB01406 (Danazol)	1.569	26.39
DB00426 (Famciclovir)	0.894	0
DB00506 (Levonorgestrel)	1.488	0
DB00299 Penciclovir	0.947	0
DB00836 (Loperamide)	0.910	0
DB00909 (Zonisamide)	1.029	0
**DB01026 (Ketoconazole)**	**0.853**	**71.63**
DB00752 (Tranylcypromine)	0.965	0
**DB00641 (Simvastatin)**	**1.083**	**82.73**
DB01217 (Anastrozole)	1.011	0
DB01128 (Bicalutamide)	0.981	0
DB00357 (Dl-aminoglutethimide)	0.959	0
**DB00586 (Diclofenac, Na)**	**0.846**	**82.86**
DB00741 (Hydrocortisone)	1.115	0

Table S6. Continue…

**DB01167 (Itraconazole)**	**1.890**	**51.99**
DB01017 (Minocycline HCl)	1.048	0
DB00834 (Mifepristone)	1.080	27.16
DB00477 (Chlorpromazine HCl)	0.981	3.11
DB00499 (Flutamide)	0.949	2.83
DB00396 (17- hydroxyprogesterone)	0.961	13.01
DB00603 (Medroxyprogesterone 17-acetate)	1.079	3.20
DB01183 (Naloxone HCl)	1.051	0

Estrogen receptor experimental assay. The restriction and modification enzymes in this work were obtained from NEB. *p*-nitrophenyl α-D-galactopyranoside (PNP-α-Gal), estradiol（E2）, yeast nitrogen base without amino acids, agar, PEG3350, lithium acetate and glucose were all purchased from Sigma. The yeast expression plasmids pGADT7 and pGBKT7 were from Clontech (Palo Alto, CA). The dropout supplement free from leucine and tryptophan (-Leu/-Trp DO supplement) was bought from Takara, and Salmon Sperm DNA was obtained from invitrogen. The yeast strain AH109 was purchased from Clontech (Palo Alto, CA).

Yeast two-hybrid system-based assay

To evaluate the agonistic or antagonistic activities of the compounds, a yeast two-hybrid system for ER was constructed by yeast co-transformation with pGBKT7-ERα/βLBD and pGADT7-SRC1 according to the lithium acetate method. [R.D. Gietz, R.A. Woods, Transformation of yeast by lithium acetate/single-stranded carrier DNA/polyethlene glycol methord, Methods Enzymol. 350 (2002) 87-96] The combination plasmid pGBKT7-ERα/βLBD

(amino acid residues 301-553 of ERα and 248-510 of ERβ) and pGADT7-SRC1 (amino acid residues 613-773) was prepared as described previously (Lin et al. *J Steroid Biochem Mol Biol* **110:** 150-156.). Butyl 4-(butyryloxy) benzoate functions as a new selective estrogen receptor beta agonist and induces GLUT4 expression in CHO-K1 cells. After co-transforming the two constructs into yeast strain AH109, we successfully evaluated ER/SRC1 interactions by conducting a convenient α-galactosidase assay. Yeast transformants were incubated with either a control vehicle (DMSO) or the indicated compounds for 24 h in hERα/β agonist testing, and in antagonist assays 1 nM E2 was added. The α-galactosidase activity was then measured using *p*-nitrophenyl α-D-galactopyranoside as the substrate. [Yeast Protocols Handbook PT3024-1, Clontech] The α-galactosidase activity was calculated according to the following formula:

$$\alpha\text{-galactosidase activity [milliunits/(mL × cell)]} = \frac{\mathrm{OD}_{410} \times V_f \times 1000}{(\varepsilon \times b) \times t \times V_i \times \mathrm{OD}_{600}}$$

where t is the elapsed time of incubation (min), V_f is the final volume of assay (200 μL), V_i is the volume of culture medium supernatant added (16 μL), OD_{600} is the optical density of overnight culture, and $\varepsilon \times b$ is the *p*-nitrophenol molar absorptivity at 410 nm× the light path (cm) = 10.5 mL/μmol.
