# Supplementary material for: Prediction of Drug-Target Interactions and Drug Repositioning via Network-Based Inference
Source: PLoS Comput Biol. 2012 May 10;8(5):e1002503. doi: 10.1371/journal.pcbi.1002503 (PMC3349722; doi:10.1371/journal.pcbi.1002503)
Supplement: Table S7 — The detailed description of drug-gene-disease associations for five approved drugs (which were extracted from DrugBank and Online Mendelian Inheritance in Man (OMIM) Morbid Map on May, 2011). (PDF) [file pcbi.1002503.s013.pdf]

Table S7. Approved Drugs and Corresponding Disease and Disease Genes obtained from OMIM (May. 1th, 2011).

Drug Name	Known Association (Target)	Validated New Target	Drug Indications	Disease Name	Predict Disease Name	Disease Gene Names	Disease Gene Entrez Gene IDs
Montelukast	LTRA1; Alox5	DPP-IV	For the treatment of asthma	Asthma	diabetes	PHF11;MS4A2;ALOX5;ADRB2;PTGDR;GPR154;HNMT;IL12B;IL13;PLA2G7;SCGB3A2;TNF;SCGB1A1	51131;2206;240;154;5729;387129;3176;3593;3596;7941;117156;7124;7356
Diclofenac	COX-1; COX2; TTR	Erα; ERβ	For the acute and chronic treatment of signs and symptoms of osteoarthritis and rheumatoid arthritis.	Osteoarthritis	neuroprptective	MATN3;FRZB;ASPN;COL2A1	4148;2487;54829;1280
Simvastatin	TNFR5; ICAM1; LT-beta; CYP3A3; MAPK3; Pr; Itgb2; PPAR-alpha; PAI-1; Caspase-3; TNF; VEGF-A; BMP-2; COLXIIIA1; CCL2; IFN-gamma; IL-6; IL-8; LA; MMP-9; RHOA; HMGCR	ERβ	For the treatment of hypercholesterolemia.	Hypercholesterolemia	PD, AD, breast cancer	APOB;LDLR;PCSK9;LDLRAP1;EPHX2;APOA2;C7orf16;ITIH4	338;3949;255738;26119;2053;336;10842;3700
Ketoconazole	CYP51A1	ERβ	For the treatment of the following systemic fungal infections: candidiasis, chronic mucocutaneous candidiasis, oral thrush, candiduria, blastomycosis, coccidioidomycosis, histoplasmosis, chromomycosis, and paracoccidioidomycosis.	Candidiasis	prostate cancer, AD	*not available*	*not available*
Itraconazole	MRP1; CYP51A1	Erα & ERβ	For the treatment of the following fungal infections in immunocompromised and non-immunocompromised patients: pulmonary and extrapulmonary blastomycosis, histoplasmosis, aspergillosis, and onychomycosis.	Pulmonary disease	prostate cancer; AD; breast cancer	*not available*	*not available*
